# The potential for evolutionary rescue in an Arctic seashore plant threatened by climate change

**DOI:** 10.1098/rspb.2024.1351

**Published:** 2024-10-02

**Authors:** Anniina L. K. Mattila, Øystein H. Opedal, Maria H. Hällfors, Laura Pietikäinen, Susanna H. M. Koivusaari, Marko-Tapio Hyvärinen

**Affiliations:** ^1^ Botany and Mycology Unit, Finnish Museum of Natural History, University of Helsinki, Helsinki, Finland; ^2^ Department of Biology, Lund University, Lund, Sweden; ^3^ Research Centre for Ecological Change, Organismal and Evolutionary Biology Research Programme, University of Helsinki, Helsinki, Finland; ^4^ Nature Solutions, Finnish Environment Institute (Syke), Helsinki, Finland; ^5^ Department of Geosciences and Geography, University of Helsinki, Helsinki, Finland

**Keywords:** evolutionary potential, evolvability, G-matrix, evolutionary rescue, climate change adaptation, pollinator decline

## Abstract

The impacts of climate change may be particularly severe for geographically isolated populations, which must adjust through plastic responses or evolve. Here, we study an endangered Arctic plant, *Primula nutans* ssp. *finmarchica*, confined to Fennoscandian seashores and showing indications of maladaptation to warming climate. We evaluate the potential of these populations to evolve to facilitate survival in the rapidly warming Arctic (i.e. evolutionary rescue) by utilizing manual crossing experiments in a nested half-sibling breeding design. We estimate G-matrices, evolvability and genetic constraints in traits with potentially conflicting selection pressures. To explicitly evaluate the potential for climate change adaptation, we infer the expected time to evolve from a northern to a southern phenotype under different selection scenarios, using demographic and climatic data to relate expected evolutionary rates to projected rates of climate change. Our results indicate that, given the nearly 10-fold greater evolvability of vegetative than of floral traits, adaptation in these traits may take place nearly in concert with changing climate, given effective climate mitigation. However, the comparatively slow expected evolutionary modification of floral traits may hamper the evolution of floral traits to track climate-induced changes in pollination environment, compromising sexual reproduction and thus reducing the likelihood of evolutionary rescue.

## 1. Introduction

Ongoing rapid changes in natural habitats and climate pose serious threats to ecosystems. While some populations can and have responded through range shifts [1] such responses are not always plausible. Due to anthropogenic habitat loss, dispersal may be increasingly limited. In such a case, persistence requires sufficient potential for phenotypic adjustment in variable and changing conditions (plasticity [2]) or, across generations, evolutionary adaptation [3]. Plastic responses are unlikely to allow populations to persist under continued environmental change [4,5], and often, the only mechanism of long-term persistence may be adaptive evolutionary change [6,7]. These may not be entirely independent mechanisms, however, because plastic responses may sometimes facilitate adaptation [8,9] or restrict it [10]. Furthermore, recent studies suggest that more plastic traits are often also more evolvable [11,12].

Considering the expected rate of climate change, a valid concern is whether evolutionary adaptation will be able to keep pace with the changing conditions, thereby allowing evolutionary rescue [13,14]. If not, adaptational lag [15] and thus maladaptation with subsequent population decline and extinction will result. For example, *Arabidopsis thaliana* populations are lagging behind in their adaptive response to climate change, with genotypes from warmer climates consistently outperforming native genotypes [16]. Similar examples of natural populations exhibiting adaptational lag and maladaptation to recent climate change are accumulating (e.g. [17–19]). Furthermore, concurrent loss, degradation and fragmentation of habitats are raising concerns over decreasing population sizes [20–22] and thus reduced additive-genetic variance and evolutionary potential [23,24] (but see [25]).

On the other hand, it is increasingly clear that evolutionary adaptation can sometimes occur sufficiently fast to track changing adaptive optima (e.g. [26,27]). Rapid human-induced environmental perturbations are subjecting natural populations to strong natural selection [28], which can lead to population decline or extinction but also facilitate rapid evolution given sufficient evolutionary potential, demonstrated in a growing number of examples from natural systems (e.g. [29–31]).

Beyond knowledge about patterns of selection, a predictive understanding of the adaptive potential of populations hinges on relevant estimates of evolutionary potential, i.e. evolvability [32–34]. In the context of adaptation to environmental change, evolutionary quantitative genetics provides a useful framework for quantifying the evolutionary potential of populations. In this context, evolvability measures the rate of adaptation (rate of change in a trait due to evolution by natural selection) per strength of selection (i.e. selection gradient, which describes the relationship between a trait and relative fitness). This makes the additive-genetic variance in phenotypic traits (deviation from the mean phenotype due to inheritance of particular alleles and their relative effect on phenotype) *V*_*A*_ = Δ*z*/*β* (where Δ*z* = change in trait mean and *β* = selection gradient) a straightforward measure of evolutionary potential [35–37]. When expressed on a proportional scale through mean-standardization, evolvabilities measured as *e* = *V*_*A*_/*µ* ^2^ (where *µ* = trait mean) can be directly translated into evolutionary doubling times under a hypothesized or measured strength of selection. These doubling times can then be interpreted in relation to the expected rate of change in the relevant selective agents, such as climate, thus evaluating the potential for evolutionary rescue. Despite the intuitive appeal and high interpretability of quantitative-genetic evolvability estimates, these approaches are rarely leveraged in studies of adaptation to environmental change (but see [38]).

**Figure 1.**
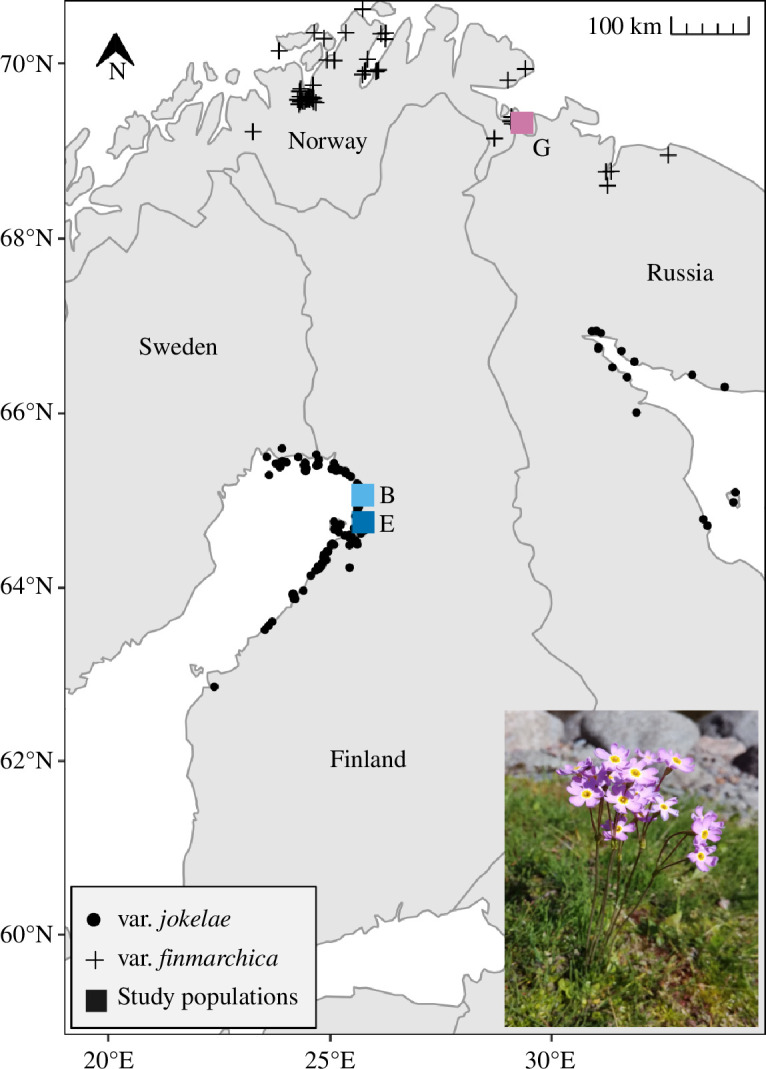
Map of the occurrence sites of the northern and southern varieties of *P. nutans* ssp. *finmarchica* and the study populations in northern Fennoscandia. Image by Anniina Mattila.

Arctic species occur at the northernmost boundaries of suitable terrestrial habitats, making them particularly sensitive to increasing temperature. Furthermore, the Arctic has warmed nearly four times faster than the globe on average during the most recent decades [39]. These factors motivate further studies of the evolutionary potential of Arctic species. Here, we study the evolutionary potential of an endangered Arctic seashore perennial herb, the Fennoscandian subspecies of the Siberian primrose (*Primula nutans* ssp. *finmarchica* (Jacq.) Á. Löve & D.). This species has a disjunct distribution separated by a distance of *ca* 400–550 km, with the northern variety (var. *finmarchica*) limited by the Arctic Ocean in Norway and the southern variety (var. *jokelae* L. Mäkinen & Y. Mäkinen) isolated from suitable habitat at the shores of the Bothnian Bay in Finland (figure 1). There is no effective gene flow between the two main areas of occurrence, reflected as genetic differentiation between the varieties [40]. A recent reciprocal transplant experiment showed that the southern variety outperforms the northern variety at the northern range edge, indicating maladaptation to realized climate change [41]. As the species is a poor disperser with specific habitat requirements [40,42], its potential to respond rapidly through range shifts is negligible. Here, we introduce a novel way of studying the potential for evolutionary rescue by integrating quantitative genetic, demographic and climatic data to estimate the potential for these primrose populations to evolve through time to keep pace with changing climate.

## 2. Methods

### (a) Study species and populations

The two studied ecotypes of *P. nutans* ssp. *finmarchica* (the northern var. *finmarchica* and the southern var. *jokelae*; figure 1) occur mainly on seashore and riverside meadows, driven by their tolerance to salty conditions and flooding in combination with poor competitive ability outside this open habitat type [42]. The mean annual temperature (1970−2000; 10 min resolution [43]) is more than 1.5°C higher in the southern distribution area [41,44]. The species can propagate vegetatively by stolons and reproduce sexually through insect-pollinated flowers. Seeds are spread via gravity, water flow and possibly birds [42,45]. Based on demographic data, the species is relatively long-lived [46]. We specifically estimated the generation time in our study area using data from Lefkovitch matrices in Björnström *et al*. [46] and the method of Bienvenu & Legendre [47]. Based on this analysis, individuals growing in optimal habitats (open seashore meadows) are estimated to have a considerably shorter average generation time (*t*_*g*_ = 3.6 yr) than those in closed habitats (average *t*_*g*_ = 7.4 yr). Thus, habitat quality may have a considerable impact on generation time, consistent with evidence indicating that sexual reproduction in *P. nutans* can markedly increase after environmental disturbance [48].

We worked with seed material collected in August and September 2012 from populations belonging to the northern var. *finmarchica* and the southern var. *jokelae*, also previously used in a translocation experiment [41]. Here, we used seeds from a subsample of those populations, all expected to be equally representative of the two varieties: two southern populations (populations B and E in Hällfors *et al*. [41]) and one northern population (population G in Hällfors *et al*. [41]; see figure 1), collected by maternal family from 57, 50 and 50 maternal individuals from populations B, E and G, respectively. The choice of populations was based on the availability of seeds from a sufficient number of mother individuals. A second northern population, population I in Hällfors *et al*. [41], was also originally chosen for the experiment, but these seeds failed to germinate. Seeds were left to dry and ripen at room temperature for a minimum of two weeks and stored in a freezer (−18 to −20°C).

### (b) Plant cultivation and crossing design

#### (i) Parental generation

From each population, we sowed 10 seeds per mother plant on 6 and 7 May 2019 at Kaisaniemi botanic garden (University of Helsinki) individually on plug trays in a 5 : 2 : 1.5 mixture of seedling peat, vermiculite and sand. From 5 to 12 September 2019, we moved the plants to the Viikki Plant Growth Facilities (University of Helsinki) and transplanted five randomly selected seedlings per maternal family to larger 0.15 l pots with a 5 : 2 : 1 mixture of soil, vermiculite and sand and kept them in a greenhouse at regularly rotated locations at 20/11°C (18/6 h light/dark). The plants were initially watered every 48 h and every 72 h starting in October and fertilized bimonthly with a 0.2% fertilizer solution (Kekkilä Professional Superex NPK 12-5-27). Biological pest control was administered as needed. Upon the development of first buds, the plants were packed under nylon tulle to prevent uncontrolled pollination.

#### (ii) Crossing design

We obtained 116 full-sibling families (54 and 62 from populations B and E, respectively), of which 105 were nested half-sibling/full-sibling families (see electronic supplementary material, tables S1 and S2) by manual crosses, where one designated sire from each maternal family (48 sires in total: 27 and 21 from populations B and E, respectively) was crossed with 1–6 randomly chosen dams (mean = 2.42 dams per sire, 34 sires crossed with >1 dam) from different maternal families within the same population. These crosses resulted in a nested full-sibling (offspring of each dam)/half-sibling (offspring of each sire) pedigree structure in the F1 generation. *Primula nutans* is distylous and within-morph incompatible. Therefore, ‘pin’ (long-styled) sires were crossed with ‘thrum’ (short-styled) dams, and vice versa. Several flowers of each dam were pollinated with pollen from the chosen sire. Unpollinated flowers were removed. Ripe seeds from the pollinated flowers (F1 generation seeds) were collected by dam in paper bags, cleaned and stored dry at room temperature. The number of flowering plants from different families was sufficient for crosses within the southern populations B and E, but not within the northern population G. Thus, for population G, we did not make crosses but only acquired trait measurements from the parental generation.

#### (iii) F1 generation

The F1 seeds were cold stratified at 6°C for four months. We then sowed five seeds per maternal family in a 5 : 2 : 1.5 mixture of seedling peat, vermiculite and sand in a greenhouse chamber (Viikki Plant Growth Facilities, University of Helsinki) at 16/6°C (18/6 h light/dark), with watering three times weekly. Starting two months after sowing, seedlings were watered every 48 h and fertilized biweekly with 0.075% fertilizer solution (Kekkilä Professional Surepex NPK 12-5-27). Three months after sowing, up to three randomly selected seedlings per maternal family were transplanted into 0.145 l pots in a 5 : 2 : 1 mixture of seedling peat, vermiculite and sand and kept in regularly rotated locations in an outdoor shading tent (Kumpula Botanic Garden, University of Helsinki) with regular mist watering and biweekly fertilization (0.1% Kekkilä Professional Turve Superex NPK 12-5-27). Biological pest control was administered as needed.

### (c) Trait measurements

#### (i) Parental generation

Upon flowering, the timing of the first open flower was recorded and a set of vegetative and floral trait measurements were taken, including (i) leaf rosette width, (ii) length and width of the largest leaf, (iii) height of the highest inflorescence from the rosette to the top of the flowers, (iv) flower corolla diameter from up to three open flowers, and (v) flower morph (‘pin’/‘thrum’). Measurements were made in spring 2020 using digital calipers (Mitutoyo Absolute AOS DIGIMATIC model CD-15APX, 0.01 mm precision).

#### (ii) F1 generation

Because the experiment was continued only until the flowering stage of the F1 offspring, the reproductive fitness of the study individuals could not be measured through their capacity to produce viable offspring, but other key plant performance measures likely to correlate with fitness [49] were recorded in spring–summer 2021. The timing of the first open flower was recorded, and when plants had at least two open flowers, a set of vegetative and floral traits were measured as in the parental generation. Additionally, the total number of flowers per individual was recorded, and the lengths of sexual organs were measured from up to three open flowers (length from the base of the corolla tube to the tip of the anthers and stigma, respectively, similarly for both ‘pin’ and ‘thrum’ type flowers). Plant individual-wise mean values were calculated for floral traits with multiple measurements per individual. The trait measurements were obtained from 1 to 14 (mean 4.06) offspring individuals of each sire (mean 1.67 offspring individuals of each dam), resulting in data from a total of 192 F1-generation individuals (in total 86 and 106 individuals from populations B and E, respectively; for details, see electronic supplementary material, tables S1 and S2).

### (d) Data analysis

#### (i) Covariance of measured traits

To study the covariance of traits in the raw F1 data, i.e. ignoring family relationships, we estimated a mean-scaled phenotypic variance matrix (P) by computing a covariance matrix of the studied traits and calculating mean P over the combinations of populations and floral morphs. For comparison, we also computed the corresponding correlation matrix of traits (Pearson correlation). To estimate the relationship of the recorded traits with reproductive fitness, we additionally calculated the correlation of traits (Pearson correlation) with the expected closest proxies of sexual and vegetative reproductive output available: the total number of flowers (expected to correlate with pollen yield and fruit set; e.g. [50–52]) and the number of stolons, respectively.

#### (ii) Quantitative-genetic analysis

To estimate the G-matrix ( G ), we fitted a multivariate animal model with the MCMCglmm R package [53] of the form


$$z_{ijk}=\;\mu_{i}+\;a_{ij}+b_{ij}+q_{ijk},$$

where *z* is the phenotypic trait value, *μ* is the trait mean, *a* is the breeding value, *b* is the non-genetic plant-level effect and *q* is the residuals. The indices run over traits (*i*), plants (*j*) and unique data points (*k*). To achieve a mean G-matrix of the populations, we pooled data from the southern populations B and E, justified by the geographical vicinity and phenotypic similarity of the populations, as well as the aim of achieving one high-quality estimate rather than two separate lower quality ones, given the available amount of data. We included floral morph (‘pin’/‘thrum’) as a fixed effect, thus estimating the variance components while accounting for any difference between morphs. This was also necessary because traits directly related to floral morph (anther and stigma length) were included in the model. The ‘animal’-level random effect was distributed as a∼N(0, G ⊗ A) , where A is the additive relatedness matrix derived from the breeding design and ⊗ is the Kronecker product, and yields the additive-genetic variance–covariance matrix G [54]. We sampled the posterior distribution for 75 000 iterations with a transient of 25 000 iterations discarded and a thinning interval of 50. We assessed and confirmed convergence by evaluating posterior trace plots and confirming effective sample sizes close to the expected 1000 posterior samples. Finally, we compared the estimated mean-scaled P and G through random skewers, i.e. by correlating the magnitudes of their expected responses to 1000 random unit-length selection gradients [55].

#### (iii) Measuring evolvability

Here, evolvability is defined as the mean-scaled additive-genetic variance, giving the expected percentage change in the trait mean per generation under a unit strength of selection (i.e. selection gradient; *β*) [50]. For multivariate phenotypes described by the additive-genetic variance matrix G , the mean evolvability *e* is given by the mean of the eigenvalues, which equals the mean of the univariate evolvabilities [55]. We also compared the multivariate autonomy (‘independence’) of trait groups (see electronic supplementary material, S4).

To facilitate interpretation, we used the evolvability estimates of a subset of the G-matrix traits (those available from the parental generation) to calculate the time (in generations) it would take to evolve from the northern phenotype to the southern phenotype under two different selection scenarios: (i) under strong selection (mean-scaled *β* = 1 = the strength of selection on fitness as a trait) and (ii) under average selection strengths for comparable floral and vegetative traits from a recent meta-analysis [56]. We calculated the times as


$$t=\frac{\ln\left(z_{S}/z_{N}\right)}{e\beta },$$

where $z_{S}$ is the southern phenotype (expected new phenotype of the northern variety following climate-driven evolution = current phenotype of the southern variety), $z_{N}$ is the northern phenotype (ancestral phenotype of the northern variety), e is the evolvability (mean-standardized additive-genetic variance *e* = *V*_*A*_/*µ* ^2^, in which *V*_*A*_ is the variance associated with the ‘animal’-level random effect from the estimated multivariate additive-genetic variance matrix G , as described above, and *µ* is the trait mean) and β is the mean-scaled selection gradient [51].

#### (iv) Divergence analysis

First, we compared phenotypes in the northern and southern populations. We then asked whether the populations have diverged in a direction in phenotype space with greater-than-average evolvability. This was done by comparing evolvability along the divergence vector from the northern population to the pooled southern populations with the mean evolvability of the compared traits, i.e. the evolvability in a randomly chosen direction in phenotype space. We defined the divergence vector as $\Delta {\underline{x}}_{NS}=\log\left({\underline{x}}_{N}\right)-\log\left({\underline{x}}_{S}\right)$ , where ${\underline{x}}_{N}$ denotes the vector of northern trait means and ${\underline{x}}_{S}$ the vector of southern trait means, and computed the evolvability along this vector scaled to unit length as $e(\Delta {\underline{x}}_{NS})$ = ${\Delta {\underline{x}}_{NS}}^{T}$ $G\Delta {\underline{x}}_{NS}$ , where *T* denotes transposition. A caveat of this analysis is that we could not estimate evolvability for the northern populations, so the divergence analysis assumes that the northern population G-matrix is similar to that of the southern populations. The stability of **G** is unresolved. It has been previously suggested that, for esample, range-limit populations may differ from distributional core areas in genetic diversity and that this could be reflected also in their levels of evolutionary potential, but to this end, there is limited empirical evidence [57]. Some studies indicate that **G** is evolvable and may change rapidly through generations and populations (e.g. [58]). However, a problem in this field is that G-matrix comparisons have been done using a diversity of methods and sometimes with questionable choices such as analyses of variance-scaled data, which tend to obscure effects [36]. On balance, especially for morphological traits, it is reasonable to assume that G-matrices are quite stable within species [59–61].

Our study system was designed to optimize quantitative genetic data, but generational differences in growth conditions (greenhouse versus shade house) allowed us to also evaluate patterns of plasticity (for further information, see electronic supplementary material, S3).

#### (v) Climatic data

To set the evolvability values into the context of climatic variables and expected rates of climate change in the study population regions, we extracted climatic data from the WorldClim database (CMIP6, 10 min resolution, mean calculated over MIROC6, GISS-E2-1-G, MPI-ESM1-2-HR, BCC-CSM2-MR, MRI-ESM2-0 models for SSP126/‘1.5°C rise’ and SSP585/‘worst-case/no-policy’-scenarios [43]; about the choice and extraction of climatic data, see electronic supplementary material, S5).

## 3. Results

**Figure 2.**
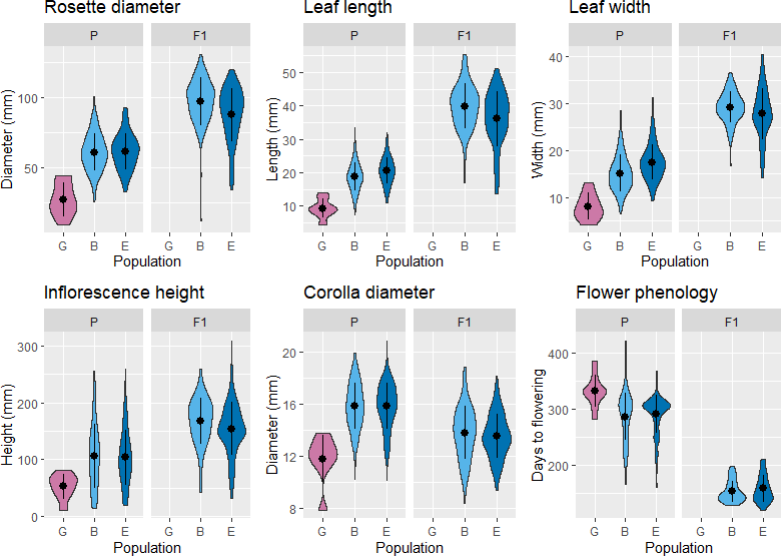
Distributions of vegetative and floral traits in the *P. nutans* study populations G (north; purple), B (south; light blue) and E (south; dark blue) in the parental (P; cultivated in greenhouse) and offspring (F1; cultivated in shade house) generations. Black dots represent the mean values, and the black lines represent s.d.

The means of all tested traits differed between the northern (population G) and southern (populations B and E) parental populations and more so in vegetative traits compared to flower size and phenology (figure 2 and table 1; statistical test results are given in electronic supplementary material, table S6). For instance, populations differed in rosette diameter by 77%, while they differed in flower corolla diameter by only 29% and in flowering phenology by 14%. In contrast, the two southern populations had very similar trait means (figure 2 and table 1; see electronic supplementary material, table S6, for formal testing). To simplify interpretation, we chose only one leaf size trait (leaf length) for subsequent analyses.

**Table 1.** Trait value means in parental (P) and offspring (F1) generations of study populations B (southern; S), E (southern; S) and G (northern; N).

generation	population	rosette diameter (mm)^a^	leaf length (mm)^a^	leaf width (mm)	inflorescence height (mm)^a^	number of flowers	number of stolons	anther length (mm)	stigma length (mm)	corolla diameter (mm)^a^	days to flowering^a^	*n*
P	G (N)	27.11	9.30	8.09	53.08	3.13				11.80	332.09	8
P	B (S)	61.04	18.66	15.19	106.23	7.01				15.84	286.40	158
P	E (S)	61.68	20.55	17.53	104.17	7.06				15.82	290.71	134
F1	B (S)	97.38	39.85	29.22	168.38	5.84	0.95	6.85	6.79	13.80	152.64	86
F1	E (S)	87.78	36.07	27.81	154.42	6.10	1.07	6.35	6.77	13.54	157.74	106
percentage difference in S versus N populations		77.4%	70.9%	67.1%	65.9%	76.9%				29.2%	14.1%	
percentage change in F1 versus P generations		50.2%	93.6%	74.9%	52.6%	−15.0%				−13.7%	−46.1%	

Two bottom rows show the percentage difference in trait mean values between the southern (pooled B and E) and northern populations in the parental generation and the percentage change in trait mean values in the F1 generation compared to the parental (P) generation (pooled B and E populations).

^a^ Denotes traits included in the divergence analysis.

The phenotypic variance matrix (**P**; table 2) showed that the studied traits can be grouped into the following three modules: (i) ‘vegetative traits’ (rosette diameter, leaf length and inflorescence height) and flowering phenology (negatively correlated with growth, classified henceforth as a ‘life-history trait’), (ii) ‘flower size traits’ (corolla diameter), and (iii) ‘flower-pollinator fit traits’ (anther and stigma length). Floral trait groups 2 and 3 together are henceforth referred to as ‘floral traits’. These trait groups were correlated within modules, but less so among modules. Vegetative traits were most highly correlated with the expected closest proxy of sexual reproductive output available, the total number of flowers and also positively correlated with the number of runners, a proxy of vegetative reproductive output (electronic supplementary material, table S7).

**Table 2.** Phenotypic covariance matrix **P** of *P. nutans* vegetative and floral traits.

	rosette diameter	leaf length	inflorescence height	anther length	stigma length	corolla diameter	days to flowering
rosette diameter	**3.889**	3.113	1.223	0.451	0.358	0.089	−1.328
leaf length	0.788	**4.012**	1.499	0.385	0.250	0.143	−1.278
inflorescence height	0.230	0.277	**7.284**	0.309	0.362	−0.141	−1.061
anther length	0.208	0.175	0.104	**1.210**	0.839	0.509	−0.558
stigma length	0.159	0.109	0.117	0.667	**1.308**	0.278	−0.558
corolla diameter	0.033	0.052	−0.038	0.337	0.177	**1.887**	0.081
days to flowering	−0.492	−0.466	−0.287	−0.370	−0.356	0.043	**1.875**

Values on and above the diagonal show the mean-scaled phenotypic covariance matrix **P**, scaled by trait mean and given as %. Values below the diagonal show the corresponding correlation matrix (Pearson correlation).

Turning to the additive-genetic variance matrix (**G**; table 3), the evolvability of vegetative traits was 10 times higher than the evolvability of floral traits. The mean evolvability of vegetative traits (*e* = 2.51%) and of floral traits (*e* = 0.24%) translates into evolutionary doubling times of 28 and 287 generations, respectively, assuming unit strength selection (*β* = 1; table 4). The G-matrix confirmed positive genetic correlations among the vegetative traits, with the exception of inflorescence height, which was largely independent. Furthermore, vegetative traits are largely decoupled and hence modularly independent from floral traits, flower size is decoupled from flower-pollinator-fit traits and flowering phenology is genetically largely independent (table 3). Autonomy (*a*) values also support such modularity: the autonomy between the vegetative and floral trait groups is very high (*a* = 0.99) and higher than the multivariate autonomy within each group (*a*_Vegetative_ = 0.87, *a*_Floral_ = 0.94). Finally, the phenotypic and additive-genetic variance matrices were correlated with each other (*r* = 0.718).

**Table 3.** Genetic variance matrix (**G**) of *P. nutans* vegetative and floral traits (posterior median values).

	rosette diameter	leaf length	inflorescence height	anther length	stigma length	corolla diameter	days to flowering
rosette diameter	**2.640**	1.850	−0.512	0.063	0.017	0.066	−0.270
leaf length	0.710	**2.572**	−0.300	0.051	−0.001	0.125	−0.235
inflorescence height	−0.207	−0.123	**2.313**	−0.006	−0.011	−0.055	0.243
anther length	0.087	0.071	−0.008	**0.201**	0.072	0.004	−0.081
stigma length	0.026	−0.002	−0.018	0.400	**0.160**	0.001	−0.053
corolla diameter	0.067	0.129	−0.060	0.016	0.005	**0.366**	0.004
days to flowering	−0.267	−0.235	0.256	−0.290	−0.211	0.010	**0.389**

Upper and lower diagonal sections give the covariance and correlation matrices, respectively.

Trait-specific evolvability values are shown on the diagonal.

**Table 4.** Mean evolvability (*e*) estimates of *P. nutans* vegetative traits (rosette diameter, leaf length and inflorescence height), floral traits (anther length, stigma length and corolla diameter) and life-history traits (flowering phenology).

traits	*e* mean	doubling time (generations; *β* = 1)^a^	doubling time (years; *β* = 1)^b^	*e* min^c^	*e* max^c^	*e* median^d^
all	1.234	56.5	203–418	0.105	4.647	
vegetative	2.508	27.9	100–206	0.741	4.602	2.08
floral	0.242	286.8	1032–2122	0.106	0.366	0.98
life-history	0.438	158.6	571–1174	0.000	1.579	

^a^ Doubling time (generations) given *e* mean and *β* = 1.

^b^ Doubling time in years given *e* mean, *β* = 1 and estimated generation time of *P. nutans* (*t*_*g*_ = 3.6–7.4 yr).

^c^ Minimum and maximum evolvability values of **G** according to Hansen & Houle [55].

^d^ Median evolvability values of vegetative and floral traits from a meta-analysis of 54 plant taxa [33].

### (a) Divergence analysis

The evolvability along the divergence vector between the northern population and the two southern populations (*eΔ*_SN_ = 3.231%) was two times as high as the mean evolvability of the **G** estimated for the southern populations ( $\overset{-}{e}$ = 1.656%). Thus, the populations diverged in a direction of greater-than-average evolvability (*eΔ*_SN_ / $\overset{-}{e}$ = 1.952).

Under a scenario of selection as strong as selection on fitness as a trait, vegetative traits could evolve from the northern population mean to the southern population mean in as little as 29 or 31 generations (leaf length and rosette diameter, respectively; table 5). If we instead assume the median strength of selection on plant size from Opedal [56], the times increase to 76 or 81 generations. In comparison, evolving from the northern to the southern mean flower corolla diameter would take 80 generations under unit strength selection and up to 217 generations under the median strength of selection on flower size (table 5). Because we lacked measurements of sexual organs in the parental generation, we could not estimate divergence in these pollinator-fit traits.

**Table 5.** Time in generations to evolve from the northern phenotype (population G) to the southern phenotype (combined populations B and E), assuming evolvability values of the estimated G-matrix (table 3).

assumed selection strength	rosette diameter	leaf length	inflorescence height	corolla diameter	days to flowering
*β* = 1 = selection on fitness as a trait	30.9	28.8	29.6	80.2	36.3
*β* = 0.37 for floral traits, *β* = 0.38 for plant size	81.4	75.9	77.9	216.9	98.2

### (b) Evolvability estimates in the context of climate change scenarios

The difference in the historical average (1970–2000) of mean temperature of the warmest quarter (WorldClim data, BIO10, denoted here as MTWQ) between the sampling areas of southern and northern populations (all sampled locations in Hällfors *et al*. [41]) was 3.8°C. Future climatic projections for the northern site give an estimated increase in MTWQ from 10.0°C in 1970–2000 to 12.3°C (SSP126/‘1.5°C rise’ scenario) or as high as 14.8°C (SSP585/‘worst-case/no-policy’ scenario) in 2061–2080. From this, we can retrieve an estimated rate of change of MTWQ at 0.027–0.056°C per year (assuming linear change within time period 1985–2070 = 85 years). With this rate of change, the northern site should reach the historical MTWQ conditions of the southern site in 70–140 years (in 2055–2125), which equals 9–39 primrose generations (given estimated generation time of *P. nutans t*_*g*_ = 3.6–7.4 yr).

Vegetative traits were positively correlated with total flowering output and stolon number, supporting their relevance for overall fitness. Assuming similar trait-specific evolvabilities in northern populations as those estimated here for the southern populations, the evolvability of vegetative traits implies that the northern population could evolve, e.g. leaf length equal to the southern phenotype in 29 generations under consistently strong selection (*β* = 1). Given estimated primrose generation times, this would equal 104–215 years, which is slower than the expected rate of change in MTWQ under worst-case climate scenarios, but potentially within the limits of effective tracking of the changing optimum and, thus, evolutionary rescue if climate warming can be limited to below 1.5°C. In contrast, for the northern population to evolve, mean flower corolla diameter corresponding to that of the southern population would require 80 generations or 288–592 years of consistently strong selection. Within this time, climate in the northern locations will have warmed well past the conditions historically experienced by the southern population even given optimistic climate scenarios. Similarly estimated, flowering phenology could evolve fast enough to match phenology in the southern populations only under the most optimal circumstances (consistently strong selection in an optimal habitat under the most optimistic climate scenario).

Another way to compare the estimated evolutionary rates to climate projections is to look at the estimated evolvabilities and doubling times of the studied traits. The average doubling time across the studied traits was estimated at 57 generations under strong selection (*β* = 1), equalling 205–422 years, during which MTWQ would rise by more than 5.5°C. We can also estimate the change of traits per degree rise: a 1°C rise in MTWQ would, in the worst case, only take an estimated 18 years (equalling 2.4−5.0 primrose generations, assuming generation time between 3.6 and 7.4 yr). In that time traits could evolve under strong selection (*β* = 1) by on average 3.0–6.3% (calculated using equation ${\left[\left(1+e\right)\beta \right]}^{t}$ , for example, ${\left[\left(1+0.01234\right)\right]}^{5.0}=6.3\%$ , assuming estimated mean evolvability *e*_mean_ = 1.234% and time in primrose generations *t* = 5.0).

## 4. Discussion

### (a) Quantifying the evolutionary potential of the Siberian primrose

The estimated evolvabilities imply that all the considered traits harbour substantial additive-genetic variance allowing detectable response to selection over ecologically relevant time frames (say, 10 years). It should be noted that realized evolvabilities at the occurrence sites may differ from those estimated here, as the experimental growing conditions could expose (genetically based) trait variation not evident at the home sites [62]. Compared to the median vegetative-trait evolvability from a survey of 54 plant taxa (2.08% [33]), our results suggest comparatively high evolutionary potential in vegetative traits (mean = 2.51%). With this evolvability, vegetative trait values could change via evolution on average 2.51% per generation per unit strength of selection and thus double or half in only 28 generations under selection as strong as selection on fitness (*β* = 1) [63]. Therefore, despite the small size and geographic isolation of our study populations, they exhibit substantial evolutionary potential of vegetative traits. In contrast, we estimated 10-fold lower evolvability in floral traits (mean = 0.24%), which is less than the median for these trait categories in other studied plant populations (0.98% [33]). This indicates relatively limited evolutionary potential. For example, doubling flower corolla diameter would require strong selection (*β* = 1) maintained over 190 generations. Because the primrose is a perennial species with estimated generation time of 3.6–7.4 yr, these rates translate into a substantially higher numbers of years.

The rate of adaptation will also depend on the strength of phenotypic selection, which is expected to be affected by climate change [64–66]. It is known from a previous translocation study that both growth and flowering of the study species are influenced by climate, with a negative effect of temperature on the performance of both the southern and the northern variety [41]. With the temperature effect being even stronger in the northern variety and the overall higher performance of the southern variety at the northern sites, there is compelling evidence that climate change is exerting considerable selection on both vegetative and floral traits of the Siberian primrose.

Given the greater evolvability of vegetative than of floral traits, a given strength of selection yields greater expected evolutionary change in vegetative traits [67]. The assumption of similar strength of selection may not be justified, however, because the selective agents driving adaptation in floral versus vegetative traits, and thus patterns and strength of selection, are likely different. For vegetative traits, patterns of selection may most often relate to abiotic factors such as temperature and precipitation [65,68]. For example, some studies have indicated selection for increased biomass with increasing temperature (e.g. [67]), whereas others have found decreases in growth with increased duration of heat waves [69]. The southern primrose populations exhibited considerably higher trait values in all the measured vegetative traits compared to the northern population, consistent with Hällfors *et al.* [41], in which multiple populations from both occurrence areas were investigated [41]. The known effect of climate and temperature on growth in the study species supports the expectation that the increased growth and biomass are an adaptation to the southern (warmer) environment. The observed high evolvability in these traits, assuming similarity of the G-matrix across the distribution, could allow rapid evolution towards optimal size. This could be further enhanced by the positive association of vegetative traits with both sexual and vegetative reproductive output (flower and stolon abundance).

Sexual reproduction, on the other hand, is essential for allowing the recombination of genetic material and for promoting the maintenance of genetic diversity, thus enhancing the capacity for evolutionary modification in all traits [70,71]. Although both abiotic and pollinator-mediated selection act on flowers [71], pollinators are generally considered most important [56,72]. Evolution of flowers can be influenced by pollinator community composition and abundance and the matching of flower and pollinator phenology. Pollinator-mediated selection may be particularly strong in the event of local pollinator community change, speeding up evolution compared to the selection scenarios considered above and in part balancing out the low evolutionary potential observed in floral traits [56,73]. Although we do not have explicit data on pollinator-mediated selection or current pollinators of the study species, it is known that pollination is essential for sexual reproduction in the Siberian primrose because of its distyly: the exchange of pollen between the two morphs is required for fertilization and seed production [74]. Arctic and alpine biomes generally have relatively poor pollinator service (low abundance and diversity of pollinators, limited flying time and flower visitation rates), and selection is expected to favour traits that either increase selfing rates (reduced anther–stigma separation) or increase flower attractiveness to pollinators (e.g. increased flower size) [75,76]. Given persistence of distyly, the latter is expected to be an important component of current floral selection in the primrose. The G-matrix estimated here for the primrose includes traits related to plant size as well as floral traits related to pollinator fit (anther and stigma length), pollinator attraction (corolla diameter = flower size) and phenology. The relevance of these traits for pollinator-mediated selection is also supported by meta-analysis data: plant traits subject to the on average strongest pollinator-mediated selection include traits particularly associated with (in descending order) floral display, pollinator fit, plant size, flower size and phenology [50]. Furthermore, the comparatively low evolvability of many floral trait groups seems to be a consistent trend across species and is thought to reflect historical pollinator-mediated stabilizing selection shaping the variational properties of particularly those floral traits closely associated with the mechanics of pollen transfer [33], thus supporting the role of pollinator-mediated selection in the evolutionary history of the primrose.

However, of more relevance in the present study context than the past or current status of pollinator-mediated selection pressures is evaluating how the species will be able to respond via evolutionary modification to possible future (climate-induced) change in those pressures. Global pollinator decline is a concerning trend, in which climate change plays a significant part [75] by impacting pollinator species and communities [77–79] and plant–pollinator interactions [80,81]. Many pollinator species are shifting their ranges poleward or upwards, whereas others, including bumblebees, are failing to do so and their distributions are, instead, shrinking [82]. Climate change is also linked to increasing phenological mismatching of plants and their pollinators [81,83]. Thus, climate can influence natural selection on floral traits via changes in pollinator communities [64]. Pollinator dependence is a general phenomenon also in Arctic and subarctic alpine plant species, despite the common occurrence of mixed mating systems [84,85]. In fact, pollinator-mediated impacts may become even more pronounced in the rapidly warming Arctic habitats [79,86]. In this regard, with evolutionary doubling time of primrose floral traits exceeding 100 generations even with strong selection, the potential for evolution to respond to pollinator-mediated selection to maintain sexual reproductive fitness in a changing climate may be considerably compromised. Consistent with previous studies, the evolvability of floral traits was comparatively low, while even somewhat lower in the primrose compared to median evolvability of flower size for other studied plant populations [33]. In addition to having low evolvability, restricted plastic responses in flowers (at least flower size) may further limit the potential of floral traits to respond to direct and indirect effects of climate variability [2] (see electronic supplementary material, S3). Because of their low evolvability, evolutionary response of flowers may generally, not only for the primrose, be a restricting factor in plant adaptation if climate change leads to differential pollinator-mediated selection due to changes in pollinator assemblages.

### (b) Vegetative and floral traits can evolve independently from each other

Our estimated G-matrix revealed mostly positive genetic correlations within vegetative and floral modules, which can increase the rate of adaptation when selection favours increasing or decreasing values of all traits [87]. In contrast, we estimated weak genetic correlations and high autonomy between floral and vegetative traits, which means that natural selection on either trait group will have a limited influence on the evolution of the other. For example, stabilizing selection on floral traits would not dramatically reduce the rate of evolution for, e.g. rosette size. This genetic decoupling of floral and vegetative traits should also allow flexible evolutionary responses to environmental change, which could be advantageous for the possibly maladapted primrose populations [41].

The pattern of decoupled floral and vegetative traits appears to be common in plants and is consistent with the ‘Berg hypothesis’, which posits that such decoupling is due to pollinator-mediated canalizing selection on flowers [88–90]. Primrose flower size was its own ‘module’, evolving independently from both vegetative and pollinator-fit traits, consistent with differential selection pressures on floral traits not directly involved in the mechanics of pollen transfer [33,56,91]. Besides indicating a role of pollinator-mediated selection in the evolutionary history of the primrose, our results support and highlight the general importance of pollinator-mediated selection in the evolution of floral traits.

### (c) Is evolutionary rescue possible?

Evolvability estimates can be informative for evaluating the potential for evolutionary rescue, but they do not tell the complete story. In addition to genetic factors (standing genetic variation and the introduction of novel genetic material by de novo mutation and immigration), the probability of evolutionary rescue is influenced by demographic and extrinsic factors, such as initial population size, the level of maladaptation, density-dependent and dispersal-associated effects, rate of environmental change and biotic interactions [14,92]. In general, rapid environmental change will lead to a high level of maladaptation and reduce the probability that adaptive evolution will be able to restore positive populations trends [13,14,93]. In the studied primrose populations, a population decline presumably accelerated by maladaptation [41] may already have driven these populations below a threshold leading towards extinction faster than a potentially counteracting evolutionary response [94]. However, in a demographic study of southern *P. nutans* populations, Björnström *et al.* [46] found that most populations were relatively stable (except for some small clearly declining ones) and that the importance of sexual reproduction for population growth at the local level was negligible. In the light of the present results, the reduced role of sexual reproduction underlines the importance of vegetative trait evolvability for the persistence of primrose populations, at least in the short term. The indicated possibly rapid evolutionary responses in growth may somewhat alleviate potential climate maladaptation. However, in the context of potential for evolutionary responses and evolutionary rescue, sexual reproduction and effective population size (rather than the number of shoots) have crucial roles [13,14,70,95]. The comparatively slow expected evolutionary rates of flowers and flowering phenology are unlikely to allow the evolution of floral traits to track climate-induced selection pressures, especially in the case of possibly very rapid changes in pollinator communities [64,79] and their phenology [83]. This would compromise sexual reproduction and reduce the likelihood of evolutionary rescue as, paradoxically, limited sexual reproduction may effectively hinder the evolution of all traits, including vegetative ones [70,71].

Besides evaluating the general potential for evolutionary modification in the primrose populations based on G-matrix data and trait-specific evolvabilities, we explicitly evaluated the potential for climate change adaptation by using the divergence of a subset of the G-matrix traits between the two populations occurring in climatically different sites as a guideline for expected variation of traits with climatic factors. We then inferred the expected time in generations it would take for the northern phenotype (indicated to be maladapted to the current climate [41]) to evolve to correspond with the southern phenotype under different selection scenarios, assuming similar G-matrices at both sites. The close to twofold higher mean evolvability along the primrose divergence vector from the southern to the northern populations compared to the overall mean evolvability (*eΔ*_SN_ / $\overset{-}{e}$ = 1.952) is consistent with the mean relative evolvability found in a recent meta-analysis (*e*_Divergence_/ $\overset{-}{e}$ = 1.62 [34]). This indicates that the two populations have diverged more in traits that are more evolvable. Accordingly, vegetative traits (high evolvability) have diverged more between regions than have floral traits (lower evolvability). Our result is in line with recent studies suggesting a positive relationship between evolvability and evolutionary divergence [34] and confirms the relevance of evolvability estimates for predicting how evolution tends to have occurred in the past and, thus, presumably, also how it may be expected to occur in the future.

Based on our data, such future projections include the expectations that vegetative trait mean in the northern population could evolve to the southern population mean in as little as 29 generations given strong selection, but floral traits would require a much longer period of evolution (e.g. 80 generations for flower corolla diameter), and these times can be multiplied manyfold given selection strengths generally recorded for plant traits [56], and considering the times in the units of years. Are these rates of evolution sufficient to keep pace with the rate of climate change in the region? Weighing our evolvability estimates against WorldClim climatic data, we could confirm that primrose evolution may act in concert with changing climate only under the most optimal circumstances: in only those traits with the highest evolvability estimates (vegetative traits and flowering phenology) and only under consistently strong selection in an optimal habitat (in which generation time is shortest). Finally and importantly, evolutionary rescue would only be possible under the most optimistic climate change scenarios, i.e. given climate policies are effective and will be able to retain warming below 1.5°C.

Although rapid adaptation to environmental changes has been documented in nature, it remains unclear how likely it is that such adaptive changes will eventually prevent population declines and extinction due to climate change [96]. Documented cases of evolutionary rescue in nature are rare, at least partly because simultaneous data of population dynamics and evolutionary changes are scarce [14,97]. The available examples are from common species that were exposed to severe anthropogenic pressures, such as anti-vitamin K resistance in rats [98] and biological control in rabbits [99]. Concerningly, recent evidence indicates that the expected rate of environmental change may outpace the rate of evolution in many natural populations [96,100]. Based on our study, this is also likely to be the case for the endangered Siberian primrose.

## Data Availability

Data and code used to conduct the analyses are openly available in the Dryad data repository [101]. Supplementary material is available online [102].
